# Clinical Characteristics During and After COVID‐19 Infection Among Healthcare Workers During the First Wave of Omicron in Chongqing, China

**DOI:** 10.1002/iid3.70141

**Published:** 2025-01-27

**Authors:** Haoling Tang, Zhiwei Chen, Tianquan Huang, Pingping Yu, Qiao Tang, Yue Qiu, Yunling Xue, Jing Tang, Nan Cai, Hong Ren, Mingli Peng, Peng Hu

**Affiliations:** ^1^ Department of Infectious Diseases, Key Laboratory of Molecular Biology for Infectious Diseases (Ministry of Education), Institute for Viral Hepatitis The Second Affiliated Hospital of Chongqing Medical University Chongqing China; ^2^ Health Medical Center The Second Affiliated Hospital of Chongqing Medical University Chongqing China; ^3^ Department of Infectious Diseases The First Affiliated Hospital of Chongqing Medical University Chongqing China

**Keywords:** COVID‐19, healthcare workers, prevention, SARS‐CoV‐2, symptoms

## Abstract

**Background:**

Revealing the clinical manifestations and associations of COVID‐19 before and after negative transition remains an area of significant uncertainty. The aim of this study is to investigate the clinical characteristics observed during and after Omicron infection among a specific population, namely healthcare workers (HCWs).

**Methods:**

From November 4, 2022, to January 15, 2023, HCWs in our hospital were enrolled to document clinical symptoms, prevention, and treatment for COVID‐19 using a structured questionnaire.

**Results:**

A total of 1101 HCWs were included, with SARS‐CoV‐2 infection detected in 78.20% (861/1101) during the observation period. The median duration for nucleic acid conversion was 8 days. Forty‐three symptoms were identified during SARS‐CoV‐2 infection (11 symptoms per individual). The common symptoms were fever, cough, headache, phlegm production, and fatigue (60.67%–83.29%). These symptoms can be further categorized into five groups: fever type, upper respiratory tract type, influenza type, digestive system type, and systemic type—all showing complex and diverse patterns. Following SARS‐CoV‐2 infection, a total of 19 symptoms were recorded including four newly emerged ones: reduced lung capacity, memory loss, lethargy, and inattention. Importantly, we observed a significant association between gastrointestinal symptoms during the nucleic acid positive phase and subsequent neuropsychiatric manifestations after negative conversion. Interestingly, females experiencing menstruation or pregnancy exhibited a higher risk of infection, while inhaled vaccines and thymosin demonstrated a protective effect against SARS‐CoV‐2 infection.

**Conclusions:**

The clinical manifestations observed in HCWs during and after Omicron infection displayed intricate patterns, shedding new light on the complex interplay between SARS‐CoV‐2 and humans.

## Graphical abstract

Our study provides a comprehensive depiction of the clinical symptoms experienced by healthcare workers during and after SARS‐CoV‐2 infection in China's Omicron wave, shedding light on the intricate interplay between these symptoms and offering novel insights into the physiological impact of SARS‐CoV‐2 on human health. Additionally, our study revealed a significantly heightened susceptibility to Omicron infection among females during menstruation or pregnancy.

## 1 Introduction

The ongoing global prevalence of the coronavirus disease 2019 (COVID‐19), caused by severe acute respiratory syndrome coronavirus 2 (SARS‐CoV‐2), remains a significant concern. As of now, COVID‐19 has resulted in over 775 million confirmed cases and claimed the lives of more than 7 million individuals [1]. After experiencing successive waves of pandemic driven by Alpha, Beta, Gamma, and Delta variants globally, the Omicron variant and its sub‐lineages have now emerged as the predominant lineage of SARS‐CoV‐2 [2]. Therefore, elucidating the clinical characteristics of Omicron infection in different regions is crucial for optimizing strategies aimed at preventing and controlling COVID‐19.

During the initial phase of the COVID‐19 outbreak, China implemented stringent measures for epidemic prevention and control, effectively averting multiple waves of diverse SARS‐CoV‐2 variants in various regions across the country, including inland cities like Chongqing. The majority of individuals remain uninfected by SARS‐CoV‐2 and have comprehensive primary and booster vaccination coverage. However, with adjustments made to China's COVID‐19 prevention and control strategies [3], a nationwide spread of SARS‐CoV‐2 infection has emerged since December 8, 2022, marked by the first wave being attributed to Omicron BA.5 variant in Chongqing [4]. Given that prior infection with SARS‐CoV‐2 and varying vaccination experiences may lead to distinct clinical symptoms and prognoses during this Omicron BA.5 outbreak wave [5], it is helpful for us to elucidate the characteristics of Omicron BA.5 infection in Chongqing, China.

Healthcare workers (HCWs) represent a relatively homogeneous subgroup with distinct characteristics. Due to the nature of their role, this group faces a potentially higher risk of infection, particularly during the early stages of a pandemic [6, 7]. Moreover, since the onset of the pandemic, HCWs have been subjected to systematic yet mandatory screening schedules for SARS‐CoV‐2 infection in China, which may help accurately depict the symptoms following viral shedding [3]. Previous studies have focused on this special population [8, 9, 10, 11]; however, the majority of these studies primarily emphasized evaluating vaccine efficacy and infection rates. The clinical manifestations of Omicron BA.5 in HCWs in Chongqing remain unclear.

In this study, we aimed to investigate the clinical manifestations during and following COVID‐19 infection among HCWs during the initial wave of the Omicron variant in Chongqing. Our objective was to assess changes in symptoms and identify potential risk factors, with the ultimate goal of identifying susceptible populations and providing valuable insights for mitigating Omicron‐induced infections.

## 2 Methods

### 2.1 Study Design and Participants

In this retrospective observational study, HCWs from The Second Affiliated Hospital of Chongqing Medical University were recruited through an online questionnaire to assess the incidence of COVID‐19 infections between November 4, 2022, and January 15, 2023. Our hospital strictly implemented a daily regimen of SARS‐CoV‐2 nucleic acid/antigen testing for all HCWs during this period to determine their infection status. The participants' demographic characteristics, vaccination and prior infection history, as well as symptoms experienced after testing positive or negative for nucleic acid/antigen were recorded using the questionnaire. This study was ethically approved by the Ethics Committee of The Second Affiliated Hospital at Chongqing Medical University (Ratification No. 97/2023) and conducted in accordance with the principles outlined in the Declaration of Helsinki. Informed consent was obtained from all participants.

### 2.2 Definition

The diagnosis of SARS‐CoV‐2 infection was established based on the detection of SARS‐CoV‐2 RNA through real‐time polymerase chain reaction (RT‐PCR) or positive identification of SARS‐CoV‐2 antigen from nasopharyngeal swabs during the specified period. Asymptomatic infection referred to a state where individuals tested positive for SARS‐CoV‐2 without exhibiting any clinical symptoms. Symptoms following SARS‐CoV‐2 infection were defined as either persistent or newly developed symptoms occurring after negative nucleic acid or antigen tests during the observation period.

### 2.3 Questionnaire

The online questionnaire was developed by our team and implemented using the Wenjuanxing platform (Changsha Ranxing Information Technology Co. Ltd., Hunan, China), as provided in the Supporting Information. Specifically, the questionnaire gathered demographic data, including gender, age, height, weight, occupation, position, lifestyle habits, and underlying health conditions. It also assessed immunization status such as vaccination history, prior infection history, and use of immune‐related medications. Furthermore, it captured infection details encompassing the onset time of infection and symptoms experienced during the infectious period along with body temperature measurements taken throughout the illness episode. The severity of symptoms and treatment modalities employed were also considered. Physical symptoms experienced post‐virus or antigen testing negative and their impact on daily life activities were also evaluated. The clinical symptomatology in this questionnaire adhered to WHO document guidelines [12].

### 2.4 Statistical Analysis

Continuous variables were presented as means and standard deviations (SDs) or median with interquartile range (IQR), while categorical variables are expressed in absolute values and percentages (%). Intergroup difference analysis was conducted using Mann–Whitney *U* test, *χ* ^2^ test, or Fisher's exact test as appropriate. Multivariate adjusted regression models were employed to estimate odds ratios (ORs) and 95% confidence intervals (CIs) for assessing the association between pre‐infection characteristics and infection. Infected individuals were classified based on symptom incidence using the *k*‐means algorithm, which generated corresponding heat maps. Univariate logistic regression analysis was used to determine correlations between symptoms during and after infection, generating a corresponding correlation chord diagram based on *p* values. All data were analyzed using SPSS 21 software and visualized using GraphPad Prism 9 and Excel. A two‐sided *p* value < 0.05 was considered statistically significant.

## 3 Results

### 3.1 Baseline Characteristics

In this study, a total of 1104 questionnaires were collected. After excluding questionnaires with incomplete information (*n* = 2) and those misfiled by patients (*n* = 1), a total of 1101 valid questionnaires were included for further analysis. The baseline characteristics of the participants are presented in Table 1. The majority of participants were female (85.56%), with a mean age of 29.8 years. Regarding occupation, nurses accounted for 53.04% of the participants, followed by medical students (32.70%), doctors (9.08%), and administrators (5.18%). Among the participants, 223 (20.25%) had underlying diseases. Almost all participants had received at least one dose of SARS‐CoV‐2 vaccine (98.27%), with an average interval time between the last doses of vaccination and infection being approximately 1 year (364.4 days). It is worth noting that 48 HCWs (4.36%) received inhalation vaccine Ad5‐nCoV from CanSino Biologics, while thymosin was administered to prevent COVID‐19 in 128 HCWs, and none used a specific antiviral drug against COVID‐19, such as Paxlovid and Azvudine, during the observation period.

**Table 1:** Baseline characteristics of HCWs with COVID‐19 infection.

	All	Uninfected	Infected	
Characteristics	(*n* = 1101)	(*n* = 240)	(*n* = 861)	*p* value
Age (years)	29.81 ± 8.10	29.33 ± 7.45	29.95 ± 8.27	0.298
Sex (%)				0.944
Male	159 (14.44)	35 (14.58)	124 (14.40)	
Female	942 (85.56)	205 (85.42)	737 (85.60)	
BMI (kg/m²)	20.91 (19.26,22.76)	20.83 (19.47, 22.58)	20.96 (19.23, 22.77)	0.697
Department (%)				0.067
Internal Medicine	488 (44.32)	122 (50.83)	366 (42.51)	
Surgery	423 (38.42)	85 (35.42)	338 (39.26)	
Emergency/Outpatient	120 (10.92)	24 (10.00)	96 (11.15)	
Auxiliary	70 (6.36)	9 (3.75)	61 (7.09)	
Operating post (%)				0.659
Doctor	100 (9.08)	26 (10.83)	74 (8.60)	
Nurse	584 (53.04)	129 (53.75)	455 (52.85)	
Staff	57 (5.18)	11 (4.58)	46 (5.34)	
Students	360 (32.70)	74 (30.83)	286 (33.22)	
Lifestyles				
Lack of sleep (%)	312 (28.34)	64 (26.67)	248 (28.80)	0.516
Irregular diet (%)	381 (34.61)	93 (38.75)	288 (33.45)	0.127
Smoking (%)	33 (3.00)	8 (3.33)	25 (2.90)	0.73
Drinking (%)	44 (4.00)	9 (3.75)	35 (4.07)	0.826
Staying up late (%)	592 (53.77)	141 (58.75)	451 (52.38)	0.08
Aerobic exercise per week (%)				0.217
＜ 1 h	551 (50.05)	130 (54.17)	421 (48.90)	
1–6 h	479 (43.51)	99 (41.25)	380 (44.14)	
＞ 6 h	71 (6.45)	11 (4.58)	60 (6.97)	
Thymosin (%)	128 (11.63)	42 (17.50)	86 (9.99)	0.001
Vaccine schedule (%)				< 0.001
Booster inactivated vaccine	599 (54.41)	94 (39.17)	505 (58.65)	
Primary inactivated vaccine	118 (10.72)	31 (12.92)	87 (10.11)	
Recombinant protein vaccine	148 (13.44)	41 (17.08)	107 (12.43)	
Inhalation vaccine	48 (4.36)	33 (13.75)	15 (1.74)	
Others	188 (17.08)	41 (17.08)	147 (17.07)	
Interval of last vaccination (days)	364.36 ± 118.12	367.54 ± 117.16	363.53 ± 118.36	0.682
Menstruation (%)				< 0.001
No	544 (57.75)	152 (74.15)	392 (53.19)	
Yes	298 (42.25)	37 (25.85)	261 (46.81)	
Pregnancy (%)				
No	890 (94.48)	200 (97.56)	690 (93.62)	0.029
Yes	52 (5.52)	5 (2.44)	47 (6.38)	
Underlying disease (%)				
No	878 (79.75)	197 (82.08)	681 (79.09)	0.308
Yes	223 (20.25)	43 (17.92)	180 (20.91)	

*Note:* Data are presented as mean (SD), median (IQR), or *n* (%).

### 3.2 Temporal Distribution and Clinical Characteristics of the SARS‐CoV‐2 Infection in HCWs

**Figure 1:**
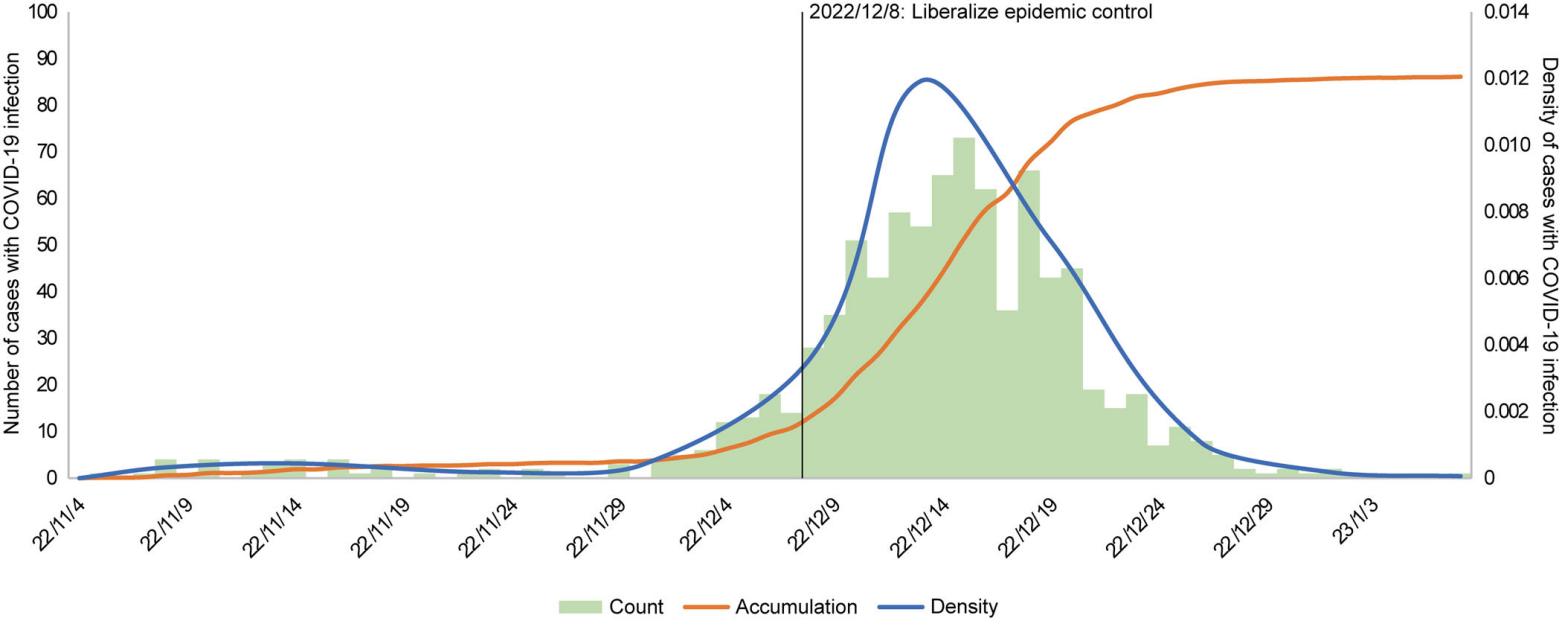
Daily incidence of COVID‐19 infection in HCWs during the first wave of Omicron infection in Chongqing (November 4, 2022, to January 15, 2023). HCWs, healthcare workers.

From November 4, 2022, to January 15, 2023, we documented the complete course of the initial Omicron wave in HCWs at our hospital, as shown in Figure 1. Specifically, COVID‐19 cases began gradually increasing from November 29, 2022—before the adjustment of COVID‐19 prevention and control measures on December 8, 2022. Subsequently, starting from December 8, there was a rapid deterioration in the situation with a peak observed by December 14. The first wave persisted for approximately 35 days and ultimately resolved by January 3, 2023.

**Figure 2:**
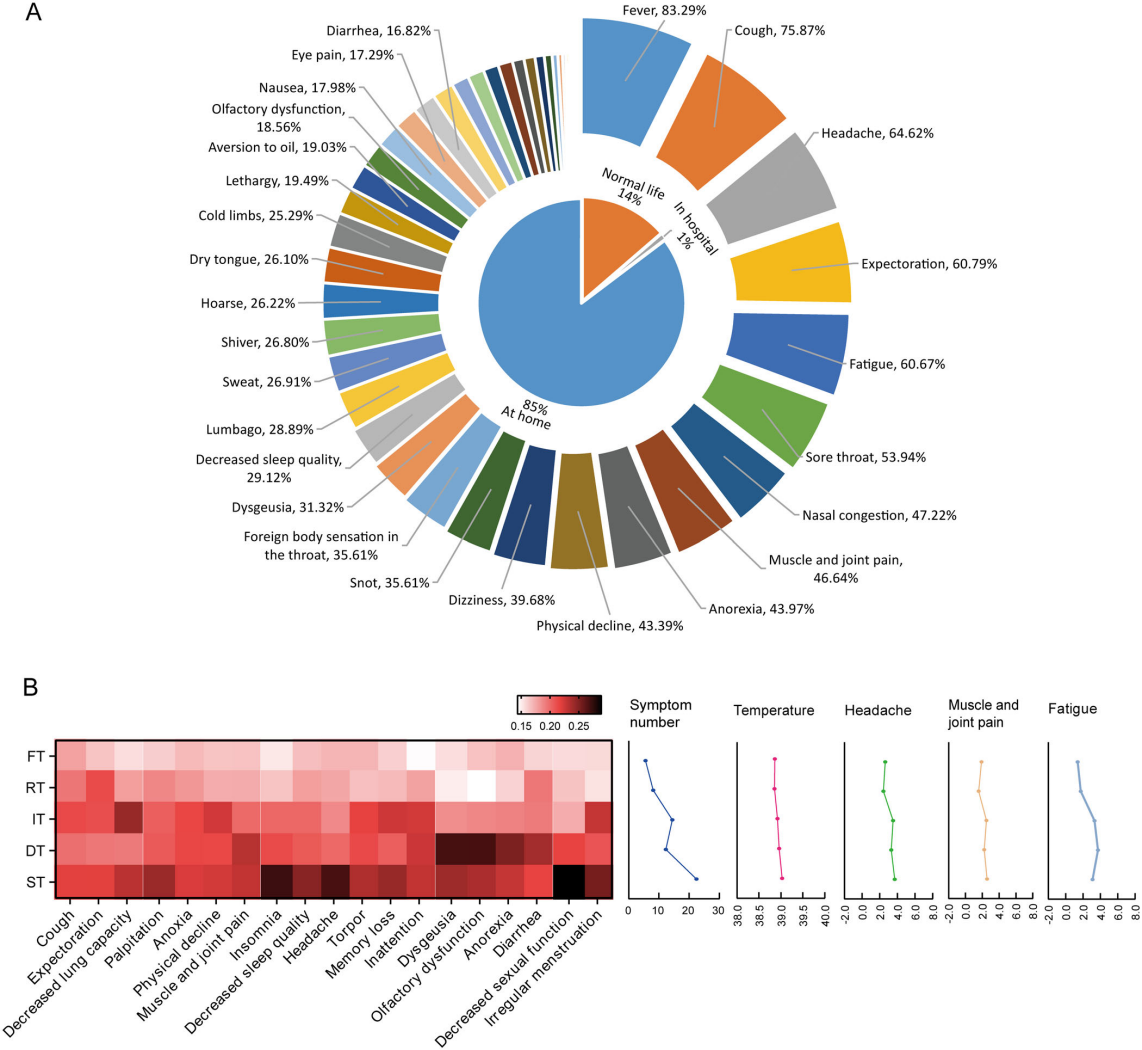
Clinical symptoms during the COVID‐19 infection in HCWs. (A) The detailed incidence of symptoms and severity in HCWs before nucleic acid/antigen negative conversion of SARS‐CoV‐2. (B) The different patterns of five symptom types and their relationship with specific features. HCWs, healthcare workers; FT, fever type; RT, respiratory type; IT, influenza type; DT, digestive type; ST, systemic type.

Throughout this observation period, SARS‐CoV‐2 infection affected a significant proportion of HCWs (861 out of a total of 1101), accounting for about 78.20%. The median duration for nucleic acid/antigen conversion was found to be 8 days (IQR 7–10 days). Among these infected cases (*n* = 861), most were classified as mild to moderate severity (99%), while only 11 required hospitalization; fortunately, no fatalities occurred during this study (Figure 2A). In addition, HCWs with underlying diseases appeared to have a higher percentage of stay at home and hospitalization; however, this difference was not statistically significant (Figure S1).

### 3.3 Clinical Symptoms During the SARS‐CoV‐2 Infection in HCWs

In this study, we documented the detailed clinical symptoms in HCWs both before and after nucleic acid/antigen conversion. Throughout SARS‐CoV‐2 infection, a total of 43 symptoms were identified, with an average of 11 symptoms per individual. No asymptomatic infections were observed in our study. The top 10 manifestations included fever (83.29%), cough (75.87%), headache (64.62%), expectoration (60.79%), fatigue (60.67%), sore throat (53.94%), nasal congestion (47.22%), muscle and joint pain (46.64%), anorexia (43.97%), and physical decline (43.39%; Figure 2A).

The population was categorized into five distinct types based on symptom cluster analysis: (i) fever type, characterized by predominantly fever symptoms with few accompanying signs; (ii) respiratory type, primarily presenting with coughing, sputum production, and other upper respiratory tract issues; (iii) influenza type, displaying influenza‐like features such as fever, headache, chills, muscle aches, and fatigue; (iv) digestive type exhibiting clear digestive system issues accompanied by mild flu‐like symptoms and possible sleep‐related discomfort; and (v) systemic type showcasing significant abnormalities throughout the entire body system (Figure 2B).

The temperature readings, symptom count, and self‐assessed scores for headache, muscle and joint pain, and fatigue exhibited complex and varied patterns among the five groups (Figure 2B). Specifically, patients in the systemic group recorded the highest temperatures, while those in the upper respiratory group recorded the lowest. Patients with systemic symptoms also displayed a wider range of symptoms compared to other groups. Headache and muscle/joint pain occurred concurrently in patients. Influenza and systemic patients experienced more severe symptoms, while those in the upper respiratory group had milder ones. Patients with digestive tract issues reported higher levels of fatigue severity, whereas those with fever exhibited comparatively lower levels.

Further, we investigated the impact of vaccination history, menstruation status, and pregnancy status on symptom patterns. No significant differences were observed in symptom patterns among pregnant women, menstruating women, and nonpregnant women (Figure S2). Notably, distinct patterns emerged between recipients of inhaled versus injectable vaccines (Figure S3). Specifically, individuals who received the inhaled vaccine predominantly exhibited digestive type.

### 3.4 Clinical Symptoms After the SARS‐CoV‐2 Infection in HCWs

**Figure 3:**
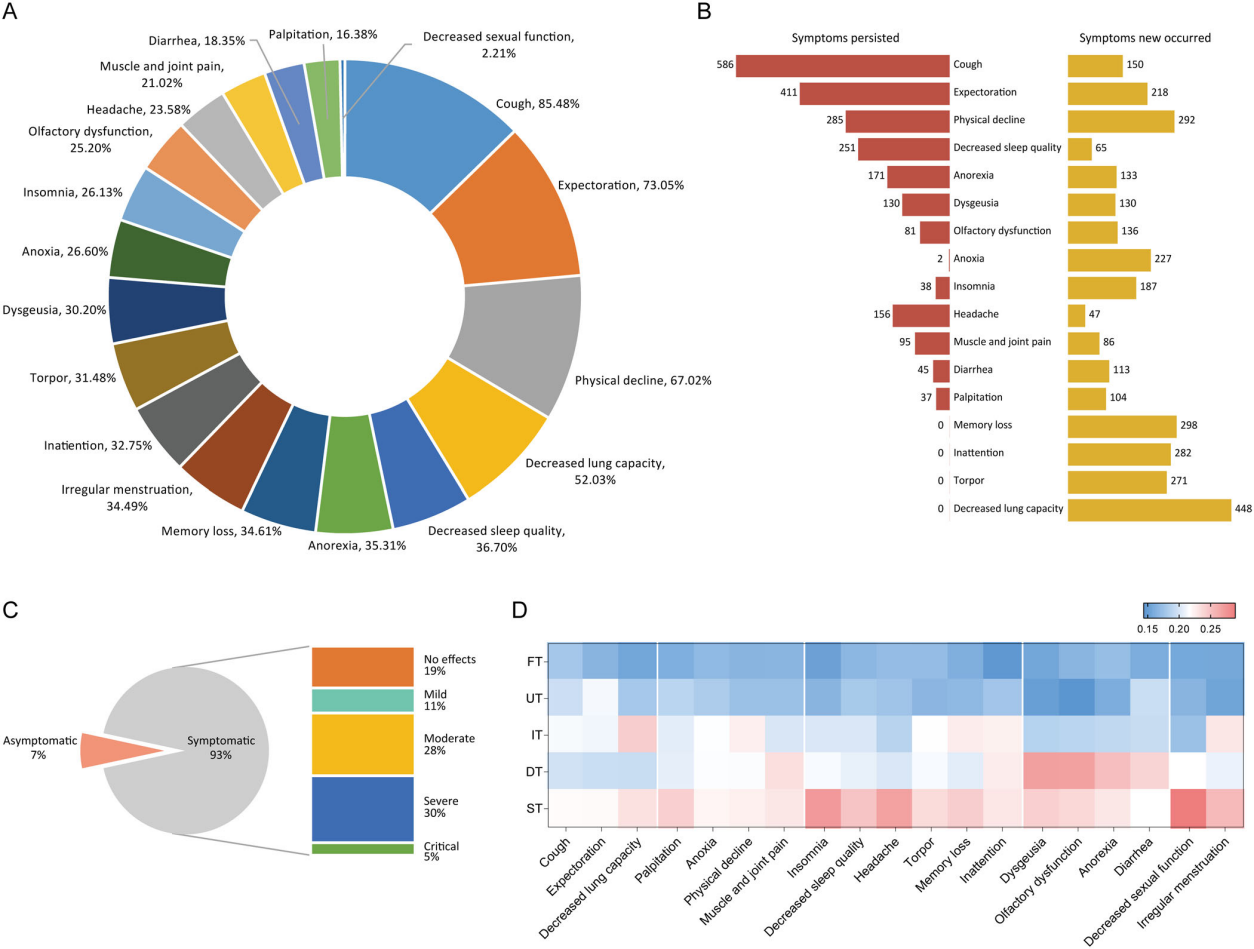
Clinical symptoms after the COVID‐19 infection in HCWs. (A) The detailed incidence of symptoms in HCWs after nucleic acid/antigen negative conversion of SARS‐CoV‐2. (B) The persisted and new occurred symptoms after infection. (C) The severity of symptoms in HCWs after infection. (D) The different patterns of five symptom types are still presented after infection. DT, digestive type; FT, fever type; HCWs, healthcare workers; IT, influenza type; RT, respiratory type; ST, systemic type.

Next, we assessed the clinical manifestations following SARS‐CoV‐2 infection. Even after negative conversion of nucleic acid/antigen, a majority of individuals (93%) remained symptomatic, with 19 different symptoms identified. Cough was the most common symptom (80%), followed by expectoration (68.52%), physical decline (58.22%), decreased lung capacity (34.38%), anorexia (30.32%), decreased sleep quality (29.51%), olfactory dysfunction (25.12%), memory loss (22.69%), inattention (22.34%), and insomnia (21%). Notably, several new symptoms emerged postinfection, such as reduced lung capacity, memory loss, lethargy, and inattention (Figure 3B). The median duration of these symptoms during observation was 17 days (IQR 13–21 days). Of note, decreased sleep quality and/or lung capacity persisted in 44% of individuals at the end of our observation period; however, compared to pre‐conversion phase, only 19% reported no noticeable impact on their daily lives (Figure 3C).

The five types of symptoms can also be accurately identified in individuals following SARS‐CoV‐2 infection, albeit with variations compared to manifestations during the nucleic acid/antigen positive stage. Influenza‐type cases primarily exhibit decreased activity tolerance and mental symptoms, while digestive tract symptoms remain predominant in cases of digestive tract type. Additionally, systemic type is characterized by neuropsychiatric symptoms and impairments in sexual function (Figure 3D).

### 3.5 Potential Interaction of Symptoms During and After SARS‐CoV‐2 Infection

**Figure 4:**
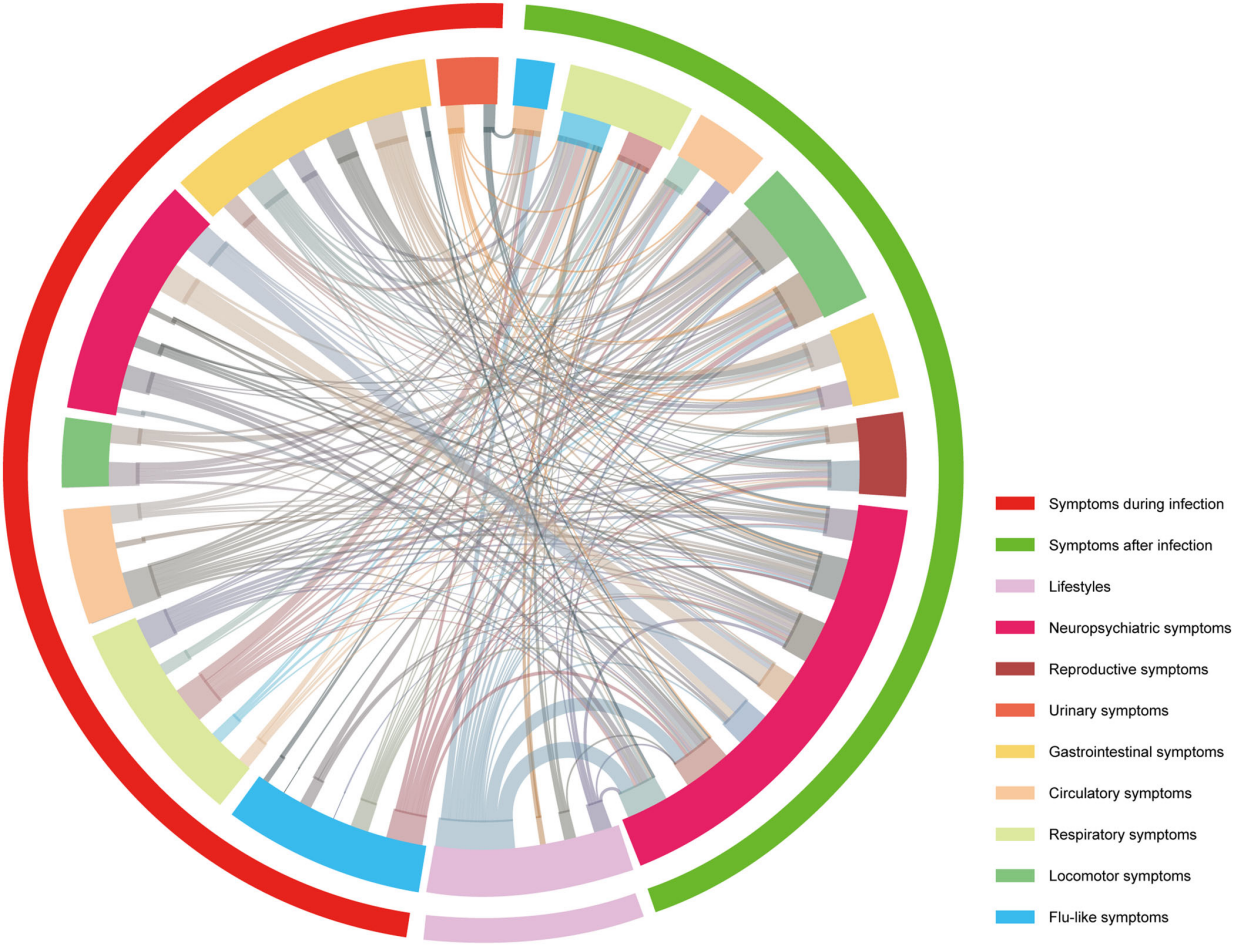
The interaction network of symptoms during COVID‐19 infection, after infection, and lifestyles in HCWs. The outermost ring represents symptoms before nucleic acid/antigen negative conversion of SARS‐CoV‐2 and lifestyles usually. The middle ring represents symptoms of the system involved. The innermost circle represents specific symptoms of each symptom. The curve represents the presence of statistically significant interactions.

Furthermore, we aim to investigate potential associations between clinical symptoms during and after SARS‐CoV‐2 infection. As depicted in Figure 4, individuals who manifest gastrointestinal symptoms during the period of nucleic acid/antigen positivity are more prone to experiencing neuropsychiatric and reproductive system symptoms following negative conversion. Similarly, individuals with reduced activity tolerance before transition exhibit a higher likelihood of developing respiratory and nervous system abnormalities post‐transition. Moreover, those presenting with respiratory symptoms and influenza‐like manifestations before conversion are more likely to persistently experience respiratory problems after negative conversion. Additionally, poor sleep habits demonstrate a positive correlation with multi‐systemic symptoms subsequent to negative conversion.

### 3.6 Risk Factors Associated With Omicron Infection in HCWs

Finally, we conducted a comparison of the clinical characteristics between HCWs with and without Omicron infection. In the univariate analysis, factors such as age, sex, BMI, department, position, lifestyles, and underlying diseases were not found to be associated with the risk of Omicron infection in HCWs (Table 1). Notably, when compared to individuals who received the booster regimen of an inactivated vaccine, those who received the inhaled vaccine exhibited a significantly lower risk of Omicron infection (OR 0.085; 95% CI 0.044–0.162). This protective effect remained significant even after adjusting for demographic and lifestyle variables (model 1: OR 0.056; 95% CI 0.023–0.133 and model 2: OR 0.052; 95% CI 0.022–0.126). Additionally, thymosin also demonstrated a significant preventive effect against Omicron infection (OR 0.523; 95% CI 0.350–0.781). Furthermore, this protective effect was more pronounced when adjusted for confounding factors (model 1: OR 0.559; 95% CI 0.325–0.962 and model 2: OR 0.544; 95% CI 0.313–0.946; Table 2). Interestingly enough, though, there was no difference observed in terms of risk between males and females regarding Omicron infection initially, but further analysis revealed that females experiencing menstruation or pregnancy showed a significantly increased risk of contracting Omicron infections (Table 2). Specifically speaking about menstruation (OR 2.387; 95% CI 1.633–3.487), this association persisted even after controlling for confounding factors (model 1: OR 4.013; 95% CI 1.190–13.528 and model 2: OR 3.640; 95% CI 1.077–12.304). The association between pregnancy and infection was more prominent as evidenced by results from both univariable (OR 2.387; 95% CI 1.633–3.487) and multivariable models (OR 3.322; 95% CI 1.986–5.557 and OR 3.289; 95% CI 1.959–5.522).

**Table 2:** Association between Omicron infection and vaccine schedule, thymosin use, and gender‐related factors.

		Univariable model		Multivariable model 1		Multivariable model 2	
		OR (95% CI)	*p* value	OR (95% CI)	*p* value	OR (95% CI)	*p* value
Thymosin use	No	1		1		1	
	Yes	0.573 (0.368, 0.891)	0.013	0.522 (0.325, 0.838)	0.007	0.521 (0.323, 0.839)	0.007
Vaccine schedule	Booster inactivated vaccine	1		1		1	
	Primary inactivated vaccine	0.514 (0.319, 0.828)	0.006	0.528 (0.324, 0.861)	0.010	0.515 (0.314, 0.844)	0.008
	Recombinant protein vaccine	0.469 (0.305, 0.722)	0.001	0.488 (0.315, 0.757)	0.001	0.488 (0.314, 0.758)	0.001
	Inhaled vaccine	0.091 (0.046, 1.051)	< 0.001	0.082 (0.041, 0.163)	< 0.001	0.077 (0.038, 0.155)	< 0.001
	Other	0.693 (0.456, 1.051)	0.084	0.729 (0.475, 1.118)	0.147	0.702 (0.456, 1.080)	0.107
Pregnancy	No	1		1		1	
	Yes	2.832 (1.101, 7.284)	0.031	3.353 (1.279, 8.792)	0.014	3.277 (1.247, 8.614)	0.016
Menstruation	No	1		1		1	
	Yes	2.683 (1.799, 4.004)	< 0.001	2.923 (1.937, 4.409)	< 0.001	2.928 (1.938, 4.422)	< 0.001
Underlying disease	No	1		1		1	
	Yes	1.340 (0.913, 1.966)	0.135	1.246 (0.839, 1.849)	0.276	1.273 (0.854, 1.898)	0.237

*Notes:* The multivariate model 1 was adjusted for age, BMI, department, and operating position. The multivariate model 2 was adjusted for lifestyle, including drinking, smoking, irregular diet, staying up late, and lack of sleep in addition to model 1.

Moreover, two risk factors were identified in relation to the duration of nucleic acid/antigen (Table 3). Firstly, vaccination status was found to be significant; specifically, individuals with primary vaccination exhibited a significantly longer duration compared to those with booster vaccination (univariable model: *β* = 1.62; 95% CI 0.336–2.865, model 1: *β* = 2.837; 95% CI 0.975–4.700, and model 2: *β* = 2.81; 95% CI 0.946–4.675). Secondly, pregnancy was associated with an extended duration of nucleic acid/antigen when infected with Omicron (*β* = 2.387; 95% CI 1.633–3.487), and this association remained significant even after adjusting for confounding variables (model 1: *β* = 2.803; 95% CI 0.346–5.259, and model 2: *β* = 2.936; 95% CI 0.467–5.406).

**Table 3:** Association between Omicron positive duration and age, vaccine schedule, thymosin use, and gender‐related factors.

		Univariable model		Multivariable model 1		Multivariable model 2	
		*β* (95% CI)	*p* value	*β* (95% CI)	*p* value	*β* (95% CI)	*p* value
Age		0.075 (0.026, 0.125)	0.003	0.078 (0.026, 0.129)	0.003	0.067 (0.012, 0.121)	0.017
Thymosin use	No	0		0		0	
	Yes	−0.421 (−1.779, 0.937)	0.544	−0.420 (−1.779, 0.939)	0.545	−0.447 (−1.808, 0.913)	0.519
Vaccine schedule	Booster inactivated vaccine	0		0		0	
	Primary inactivated vaccine	1.574 (0.190, 2.957)	0.026	1.586 (0.199, 2.973)	0.025	1.461 (0.060, 2.862)	0.041
	Recombinant protein vaccine	0.298 (−1.024, 1.620)	0.659	0.296 (−1.028, 1.619)	0.662	0.171 (−1.167, 1.509)	0.802
	Inhaled vaccine	−1.315 (−4.882, 2.252)	0.470	−1.321 (−4.891, 2.249)	0.469	−1.791 (−5.387, 1.806)	0.330
	Other	0.767 (−0.378, 1.912)	0.190	0.771 (−0.376, 1.917)	0.188	−0.608 (−0.555, 1.771)	0.306
Pregnancy	No	0		0		0	
	Yes	1.909 (0.004, 3.813)	0.050	1.874 (−0.058, 3.807)	0.058	1.866 (−0.074, 3.806)	0.060
Menstruation	No	0		0		0	
	Yes	0.083 (−0.810, 0.976)	0.855	0.077 (−0.819, 0.972)	0.867	0.049 (−0.854, 0.952)	0.915
Underlying disease	No	0		0		0	
	Yes	0.320 (−0.707, 1.347)	0.542	0.323 (−0.705, 1.351)	0.538	0.329 (−0.712, 1.371)	0.536

*Notes:* The multivariate model 1 was adjusted for age, BMI, department, and operating position. The multivariate model 2 was adjusted for lifestyle, including drinking, smoking, irregular diet, staying up late, and lack of sleep in addition to model 1.

## 4 Discussion

In this study, we comprehensively characterized the clinical symptoms preceding and following nucleic acid/antigen negative conversion of SARS‐CoV‐2, as well as identified risk factors associated with infection in HCWs during the initial wave of the Omicron BA.5 epidemic in Chongqing. The key findings are as follows: (1) we delineated five distinct symptom patterns observed in HCWs infected with Omicron, revealing intricate and interconnected relationships; (2) inhalable vaccines and thymosin demonstrated robust protective effects against Omicron infection; and (3) females experiencing menstruation or pregnancy exhibited a significantly heightened susceptibility to Omicron infection.

By leveraging the study on HCWs population, we were able to accurately and reliably collect clinical symptoms during and after SARS‐CoV‐2 infection. As anticipated, our cohort predominantly exhibited mild cases of COVID‐19, with no reported fatalities. This observation indicates a decreased virulence of the Omicron variant, even among individuals without prior SARS‐CoV‐2 infection. The common symptoms associated with Omicron in this study align with previous findings from China and other countries [13, 14]. Given that we recorded 43 symptoms of Omicron infection among 1101 HCWs, it provides an opportunity to explore potential patterns within these symptoms further. Notably, we are the first to propose categorizing these symptoms into five distinct clusters: fever type, respiratory type, influenza type, digestive type, and systemic type—each exhibiting unique and intricate patterns. Among these categories, the fever type manifests milder symptoms compared to the more severe manifestations observed in the influenza‐type cluster. The persistence of gastrointestinal symptoms suggests possible viral durability within the digestive tract or involvement of organs such as the liver responsible for digestion [15, 16, 17]. Furthermore, individuals experiencing systemic‐type symptoms appear more susceptible to neuropsychiatric and sexual dysfunction post‐negative conversion—a plausible observation considering previous studies implicating systemic inflammation in neuropathogenesis following COVID‐19 infection [18, 19]. These findings provide novel insights into the impact of Omicron infection on human physiology.

Apart from the clinical symptoms observed during SARS‐CoV‐2 infection, it is crucial to consider the manifestation and duration of symptoms after infection. Numerous studies have consistently reported persistent fatigue and dyspnea that persist for months following COVID‐19 as the most commonly reported symptoms [20]. Additionally, enduring symptoms may include cognitive and mental impairments, chest and joint discomfort, palpitations, myalgia, olfactory and gustatory dysfunctions, coughing, headache, as well as gastrointestinal and cardiac complications [21, 22]. In our study cohort, only 7% of individuals remained asymptomatic after nucleic acid/antigen negative conversion, which was lower than a previous study's findings (14.7%) [23]. This discrepancy could be attributed to differences in race, age distribution within the population studied, or lifestyle factors [24]. Furthermore, we identified several newly occurring symptoms post‐SARS‐CoV‐2 infection, such as reduced lung capacity, memory loss, lethargy, and inattention, which were also observed in previous studies [25, 26, 27]. Importantly, we also reveal an underlying interrelation between clinical symptoms during Omicron infection phase and those experienced after negative conversion. Specifically, gastrointestinal symptoms during the nucleic acid/antigen positive phase were significantly associated with neuropsychiatric manifestations post‐negative conversion. The disruption of gut microbiota by SARS‐CoV‐2 along with its impact on the brain–gut axis may contribute to this tight connection between gastrointestinal and neurological symptomatology [28, 29, 30].

Furthermore, we conducted an analysis of the risk factors associated with Omicron infection in HCWs. Consistent with previous research findings, our results demonstrate that vaccines offer substantial protection against the virus, particularly when administered via inhalation [31, 32]. Additionally, thymosin exhibits effective mitigation of COVID‐19 infection by downregulating ACE2 levels [33]. In this study, the utilization of thymosin also serves as a potent preventive measure, albeit deviating from prior research conclusions [34, 35]. Interestingly, our findings indicate that females experiencing menstruation or pregnancy exhibit heightened susceptibility to COVID‐19 infection—a phenomenon previously observed in other studies. This observation may be attributed to decreased estrogen levels during menstruation and an immunosuppressive state during pregnancy [36, 37].

Our study has several limitations. Firstly, the HCWs may not be representative of the general population; therefore, further investigation is required to determine whether similar patterns of clinical symptoms are observed in the overall population in Chongqing. Secondly, our results indicated an Omicron infection rate of 78.20%, which is consistent with findings from a previous study conducted at another hospital during the same observation period [38]. However, it should be acknowledged that some employees did not respond to this survey, which may have led to an overestimation of the infection rate in our study. Thirdly, the duration of observation for HCWs following Omicron infection was relatively short, which impeded our ability to record detailed symptoms associated with “long‐COVID‐19.” However, we did observe some symptoms persisting beyond 30 days, most of which were directly or indirectly linked to the neuropsychiatric system. Additionally, blood sampling was not conducted on individuals before and after Omicron infection in this study, preventing further analysis of the relationship between humoral and cellular immunity and the clinical symptoms and prognosis of Omicron. A well‐designed study is required to address this question in future research.

In conclusion, our study elucidated the dynamic changes in clinical symptoms observed during and after Omicron infection among HCWs, highlighting the intrinsic interplay between these symptoms, thereby offering novel insights into the impact of SARS‐CoV‐2 on human physiology.

## Author Contributions

Peng Hu and Mingli Peng designed this study. Peng Hu and Zhiwei Chen acquired the funding. Haoling Tang, Zhiwei Chen, Tianquan Huang, Pingping Yu, Qiao Tang, Yue Qiu, Yunling Xue, Jing Tang, and Nan Cai recruited participants and collected the data. Haoling Tang and Zhiwei Chen analyzed and interpreted the data. Haoling Tang and Zhiwei Chen drafted the manuscript. Hong Ren, Mingli Peng, and Peng Hu critically revised the manuscript. All authors approved the final manuscript version.

## Conflicts of Interest

The authors declare no conflicts of interest.

## Supporting information

Supporting information.

Supporting information.

Supporting information.

## Data Availability

The data that support the findings of this study are available on request from the corresponding author.
