# Supplementary material for: Clinical Characteristics During and After COVID‐19 Infection Among Healthcare Workers During the First Wave of Omicron in Chongqing, China
Source: Immun Inflamm Dis. 2025 Jan 27;13(1):e70141. doi: 10.1002/iid3.70141 (PMC11772718; doi:10.1002/iid3.70141)
Supplement: Supplementary file 1 — Supporting information. [file IID3-13-e70141-s002.docx]

**Clinical characteristics during and after COVID-19 infection among** **healthcare workers during the first Wave of Omicron in Chongqing, China**

Haoling Tang, Zhiwei Chen, Tianquan Huang, Pingping Yu, Qiao Tang, Yue Qiu, Yunling Xue , Jing Tang, Nan Cai, Hong Ren, Mingli Peng, Peng Hu

**Table of contents**

**Figure S1............................................................................................................2**

**Figure S2............................................................................................................3**

**Figure S3............................................................................................................4**


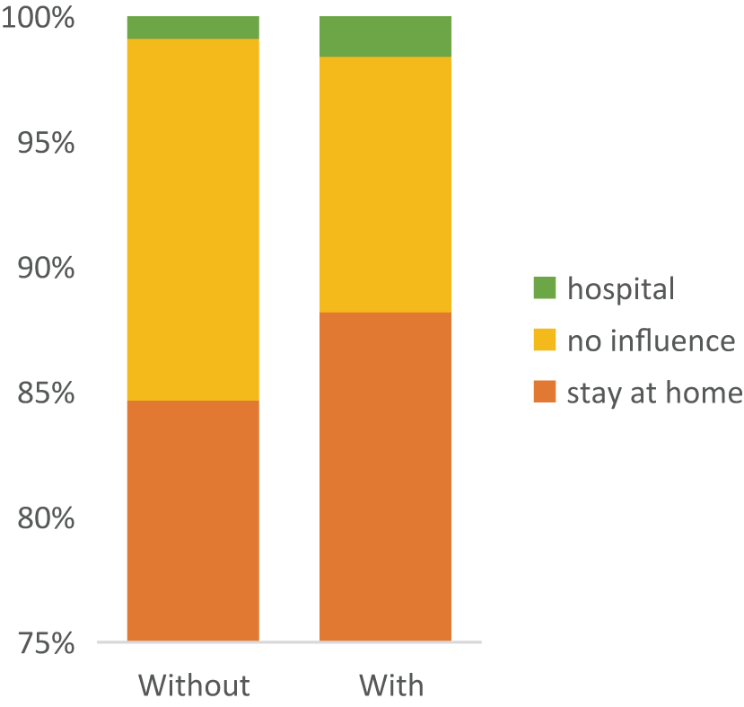


**Figure S1. The symptoms severity before nucleic acid/antigen negative conversion of SARS-CoV-2 in HCWs with and without underlying diseases.** HCWs, health care workers.


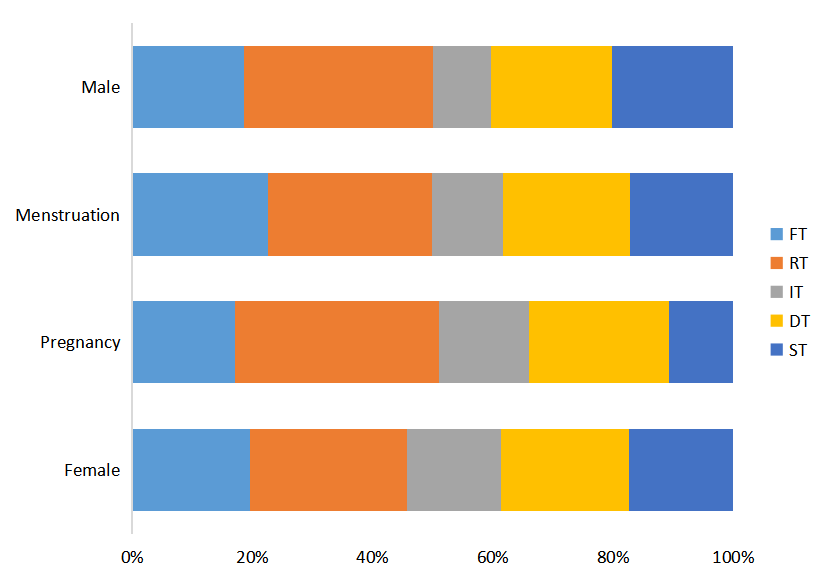


**Figure S2. The different patterns of five symptom types in HCWs with different sex, menstruation, and pregnancy statuses.** HCWs, health care workers; FT, fever type; RT, respiratory type; IT, influenza type; DT, digestive type; ST, systemic type.


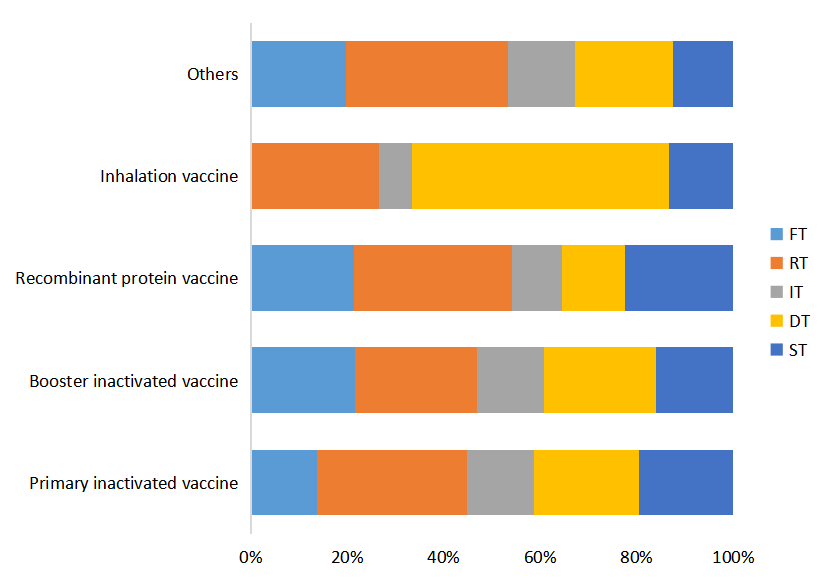


**Figure S3. The different patterns of five symptom types in HCWs with different vaccination histories.** HCWs, health care workers; FT, fever type; RT, respiratory type; IT, influenza type; DT, digestive type; ST, systemic type.
