# Supplementary material for: Clinical Characteristics During and After COVID‐19 Infection Among Healthcare Workers During the First Wave of Omicron in Chongqing, China
Source: Immun Inflamm Dis. 2025 Jan 27;13(1):e70141. doi: 10.1002/iid3.70141 (PMC11772718; doi:10.1002/iid3.70141)
Supplement: Supplementary file 2 — Supporting information. [file IID3-13-e70141-s001.docx]

## Survey on the infection situation of healthcare worker with COVID-19

*Thank you for taking the time to complete the questionnaire. It typically requires approximately 15 minutes to fill out the questionnaire. It is recommended that individuals who have not been infected or have been infected and tested negative for more than one month should complete the questionnaire.*

***We guarantee that all information from the questionnaire will only be used in this study of the Omicron epidemic, and that personal privacy is strictly protected.***

*If the question is marked with a *，the question is required.*

### Basic information

1. Name *

_________________________________

2. Age *

_________________________________

3. Phone number

_________________________________

4. Gender *

| ○ Man |
| --- |
| ○ Woman |

1. Department and operating post *

You need to choose one item in the first column and one item in the second column

| ○ Internal Medicine | ○ Doctor |
| --- | --- |
| ○ Surgery | ○ Nurse |
| ○ Emergency/Outpatient | ○ Staff |
| ○ Auxiliary | ○ Student |

6. Whether pregnant or not （Men don't need to answer）

| ○ No |
| --- |
| ○ Yes |

7. Height（cm）：such as *158 ,175* *

________

8. Weight（kg）：such as *50 ,73.5*  *

________

### Pre-infection lifestyle habits

9. Aerobic exercise time per week

| ○ ＜1h |
| --- |
| ○ 1-6h |
| ○ ＞6h |

10. Tick off the bad lifestyle habits you have [Choose at least one] *

| ○ No | ○ Smoking |
| --- | --- |
| ○ Drinking | ○ Stay up late（Sleep after 0:00） |
| ○ Lack of sleep（Sleep time＜6h） | ○ Irregular diet |

11. Sleep quality *

| ○ Terrible |  | |  |
| --- | --- | --- | --- |
| ○ Moderate | |  |  |
| ○ Great | |  |  |

### Vaccine

12. The times of vaccine shot *

| ○ 0 | ○ 1 |
| --- | --- |
| ○ 2 | ○ 3 |
| ○ 4 |  |

13. The first vaccine *

Fill in the injection time in the horizontal line，such as 21-05-12

| ○ Inactivated vaccine _________________ |
| --- |
| ○ Recombinant protein vaccine _________________ |
| ○ Inhaled vaccine _________________ |
| ○ Other vaccine _________________ |

14. The second vaccine *

Fill in the injection time in the horizontal line，such as 21-05-12

| ○ Inactivated vaccine _________________ |
| --- |
| ○ Recombinant protein vaccine _________________ |
| ○ Inhaled vaccine _________________ |
| ○ Other vaccine _________________ |

15. The third vaccine *

Fill in the injection time in the horizontal line，such as 21-05-12

| ○ Inactivated vaccine _________________ |
| --- |
| ○ Recombinant protein vaccine _________________ |
| ○ Inhaled vaccine _________________ |
| ○ Other vaccine _________________ |

16. The forth vaccine *

Fill in the injection time in the horizontal line，such as 21-05-12

| ○ Inactivated vaccine _________________ |
| --- |
| ○ Recombinant protein vaccine _________________ |
| ○ Inhaled vaccine _________________ |
| ○ Other vaccine _________________ |

### physical condition

17. Basic diseases and drug use（Fill in the drug name after the option, fill in none if not used）*

| ○ No | ○ Coronary heart disease ______________ |
| --- | --- |
| ○ Arrhythmology _________________ | ○ Hypertension _________________ |
| ○ Hyperlipemia _________________ | ○ Diabetes _________________ |
| ○ Hyperthyroidism _________________ | ○ Hypothyroidism _________________ |
| ○ Gout/hyperuricemia _________________ | ○ Anaemia _________________ |
| ○ Asthma _________________ | ○ Allergic rhinitis _________________ |
| ○ Chronic bronchitis _________________ | ○ COPD _________________ |
| ○ Tuberculosis (including previous infections) _________________ | ○ Cerebrovascular diseases: such as infarction, bleeding, etc _______________ |
| ○ Migraine _________________ | ○ Autoimmune disease ________________ |
| ○ Nephropathy _________________ | ○ Uremia _________________ |
| ○ AIDS _________________ | ○ Viral liver disease _________________ |
| ○ Fatty liver _________________ | ○ Perimenopausal syndrome ____________ |
| ○ Functional uterine bleeding __________ | ○ Tumour _________________ |
| ○ Hematological system diseases ________ | ○ Skin disease_____________ |
| ○ Neuropsychiatric disorders ___________ | ○ Other diseases _________________ |

18. Whether thymosin drugs are used for prophylaxis *

| ○ Yes |  |
| --- | --- |
| ○ No |  |

19. Whether you have been infected with COVID-19 in the past, and fill in the infection time *

| ○ Yes _________________ * |
| --- |
| ○ No |

### Information on this COVID-19 infection

20. Whether infected or not*

| ○ Yes |  |
| --- | --- |
| ○ No |  |

21. Infection date *

_________________________________

22. Whether COVID-19 has turned negative and when it has turned negative *

| ○ Yes _________________* |
| --- |
| ○ No |

23. Symptom severity *

| ○ No effect on daily activities |
| --- |
| ○ Stay at home |
| ○ In hospital（general ward） |
| ○ In hospital（ICU） |
|  |

24. Specific symptoms of infection (select at least one) *

| ○ Fever______  Fill in maximum body temperature | ○ Dysgeusia |
| --- | --- |
| ○ Cold limbs | ○ Olfactory dysfunction |
| ○ Shiver | ○ Tinnitus |
| ○ Sweat | ○ Conjunctival congestion |
| ○ Nasal obstruction | ○ Eye pain |
| ○ Snot | ○ Dry tongue |
| ○ Dyspnea | ○ Nausea |
| ○ Chest distress | ○ Vomit |
| ○ Cough | ○ Abdominal pain |
| ○ Expectoration | ○ Diarrhea |
| ○ Foreign body sensation in the throat | ○ Anorexia |
| ○ Hoarse | ○ Aversion to oil |
| ○ Sore throat ______  (Pain scale 1-10) | ○ Excessive flatus |
| ○ Muscle and joint pain ______  (Pain scale 1-10) | ○ Frequent micturition |
| ○ Lumbago | ○ Urgent urination |
| ○ Headache ______  (Pain scale 1-10) | ○ Dysuria |
| ○ Dizziness | ○ Insomnia |
| ○ Chest pain ______  (Pain scale 1-10) | ○ Decreased sleep quality |
| ○ Fatigue______  (Fatigue scale 1-10) | ○ Lethargy |
| ○ Palpitation | ○ Irritable |
| ○ Cyanosis | ○ Skin rash |
| ○ Physical decline | ○ Other symptoms ________ |

25. Whether the infection occurred during the menstrual period （Men don't need to answer）

| ○ Yes |
| --- |
| ○ No |

26. Whether the menstrual cycle or traits are normal （Men don't need to answer）

| ○ Yes |
| --- |
| ○ No |

### Treatment after this infection

27. Whether to use non-steroidal drugs, and the specific drug name *

| ○ Yes ______________ |  |
| --- | --- |
| ○ No |  |

28. Whether to use anti-COVID-19 drugs, and specific drug names *

| ○ Yes ___________ |
| --- |
| ○ No |

29. Whether other types of drugs are used, and the specific name of the drug *

| ○ Yes___________ |
| --- |
| ○ No |

### Sequelae investigation (People with negative nucleic acid transfer for at least one month fill in)

30. Weight change（such as -3kg，2kg）

_________________________________

31. Whether the following symptoms still exist after turning negative, and fill in the duration of symptoms (If you can't remember the exact time, don't fill it in) *

|  | Yes | No | Days |
| --- | --- | --- | --- |
| Cough | ○ | ○ |  |
| Expectoration | ○ | ○ |  |
| Palpitation | ○ | ○ |  |
| Anoxia | ○ | ○ |  |
| Physical decline | ○ | ○ |  |
| Decreased lung capacity | ○ | ○ |  |
| Muscle and joint pain | ○ | ○ |  |
| Insomnia | ○ | ○ |  |
| Decreased sleep quality | ○ | ○ |  |
| Headache | ○ | ○ |  |
| Dysgeusia | ○ | ○ |  |
| Olfactory dysfunction | ○ | ○ |  |
| Anorexia | ○ | ○ |  |
| Diarrhea | ○ | ○ |  |
| Decreased sexual function | ○ | ○ |  |
| Irregular menstruation | ○ | ○ |  |
| Torpor | ○ | ○ |  |
| Memory loss | ○ | ○ |  |
| Inattention | ○ | ○ |  |
| Other symptoms____________ | ○ | ○ |  |

32. The influence of COVID-19 on their normal life after it turns negative] *

| ○ Critical | ○ Severe | ○ Moderate | ○ Mild | ○ No effects |
| --- | --- | --- | --- | --- |
